# Magnesium Supplementation Improves Cortical Stratification and Neuronal Differentiation in Blood–Brain Barrier-Integrated Human Brain Organoids

**DOI:** 10.3390/biomedicines14061242

**Published:** 2026-05-29

**Authors:** Sara Castiglioni, Antonella Tosoni, Manuela Nebuloni, Jeanette A. Maier

**Affiliations:** 1 Department of Biomedical and Clinical Sciences, Università di Milano, 20157 Milano, Italy; manuela.nebuloni@unimi.it (M.N.); jeanette.maier@unimi.it (J.A.M.); 2 Division of Pathology, Ospedale Sacco, 20157 Milan, Italy; antotosoni@yahoo.it

**Keywords:** human brain organoids, magnesium, magnesium transporters, blood–brain barrier, NMDA-R, GABA-R, dopamine

## Abstract

**Background/Objectives:** Magnesium (Mg) is essential for neuronal maturation, yet its role in human cortical development remains poorly defined. Here, we investigated the effects of physiological (1 mM) and elevated (5 mM) concentrations of MgSO_4_ and magnesium pidolate (MgPid) on human brain organoids co-cultured with an in vitro blood–brain barrier (BBB) model. **Methods:** Human brain organoids derived from induced pluripotent stem cells were co-cultured with an in vitro BBB system and treated for 4 days with either MgSO_4_ or MgPid at physiological and elevated concentrations. Cortical organization was assessed by transmission electron microscopy and immunofluorescence analysis. Western blotting for neurotransmitter receptors and Mg transporters, quantification of intraorganoid Mg^2+^ levels, ELISA-based measurement of GABA and dopamine, and analysis of glutamate were performed. **Results:** High Mg exposure enhanced cortical stratification and neuronal organization, as shown by the localization of CTIP2 in the outermost layer and TBR2 in the inner layer, together with ultrastructural features consistent with advanced differentiation. Elevated Mg increased intraorganoid Mg^2+^ levels without altering Mg transporter abundance and selectively modulated neurotransmitter receptor expression: NMDA-R levels were reduced by MgPid, whereas GABA_A_-R and GABA_B_-R were upregulated, particularly in response to MgPid. Levels of glutamate, GABA, and dopamine remained unchanged. **Conclusions:** These findings identify Mg, especially in the form of MgPid, as a modulator of cortical architecture and inhibitory–excitatory receptor balance in human organoids, supporting its potential relevance for neurodevelopmental regulation and Mg-based therapeutic strategies. These results also support organoids as human-relevant, animal-free tools for neuroscience and neuropharmacological research.

## 1. Introduction

Magnesium (Mg) is an essential mineral involved in numerous physiological processes in the central nervous system (CNS), including cortical development, synaptic plasticity, and the regulation of excitatory–inhibitory balance [1]. Mg influences key excitatory and inhibitory neurotransmission pathways. It inhibits the N-methyl-D-aspartate (NMDA) receptor at the physiological membrane potential (approximately −70 mV), thus preventing excessive NMDA receptor activation, which could otherwise result in neuronal damage or death [2]. This regulation is critical during developmental windows, as NMDA-R activity governs synaptic plasticity and neuronal excitability. In murine models, maternal Mg deficiency leads to long-lasting alterations in offspring brain development, including changes in hippocampal NMDA receptor subunit expression and behavioral impairments [3]. Beyond this classical role, Mg also functions as an intracellular signaling mediator because NMDA receptor activation can promote Mg influx, triggering CREB-dependent pathways involved in neuronal plasticity and memory formation [4]. These combined effects suggest that Mg may have neuroprotective properties and could be beneficial in conditions associated with neurodegeneration [5,6].

Mg also acts as an agonist of the ionotropic gamma-aminobutyric acid type A receptor (GABA_A_-R) [7], which mediates the anxiolytic and hypnotic effects of benzodiazepines [8]. During early development, GABA_A_-R signaling triggers the release of Mg from mitochondria, leading to an increase in cytoplasmic Mg levels. This, in turn, activates the mTOR pathway, promoting neural network formation through ribosome biogenesis [2,9]. Additionally, by reshaping synaptic connectivity, intracellular Mg improves both transmission efficiency and plasticity at hippocampal synapses [10]. Of note, elevated Mg levels enhance the differentiation of primary cultured adult mouse neural progenitor cells by activating the ERK/CREB pathway while simultaneously inhibiting their glial differentiation [11], and high Mg conditions promote enteric neural crest cell differentiation by increasing glycolysis for energy production [12].

Mg also supports the integrity and function of the blood–brain barrier (BBB), a crucial cellular structure that regulates CNS homeostasis and helps preserve optimal neurological function [13]. The BBB serves as an important source of various neurotrophins, including brain-derived neurotrophic factor (BDNF), the most abundant neurotrophin in the brain, which plays a crucial role in neurogenesis, neuroplasticity, and cognitive function. Notably, Mg has been shown to increase BDNF levels [6,14].

Studies suggest that high cerebral Mg levels help reduce oxidative stress and systemic inflammation, which are factors that can impair neurogenesis and contribute to neurodegeneration [15,16,17]. Mg supplementation has been shown to decrease neuroinflammatory mediators, thereby promoting the proliferation of neuronal stem cells, particularly in the hippocampus, a key brain region involved in learning and memory [18].

Clinically, Mg, most commonly administered as magnesium sulfate, has both established and emerging uses in several CNS diseases, including eclampsia/preeclampsia [19], fetal neuroprotection, and stroke in adults [20]. In a 2026 propensity-matched cohort of 12,296 patients with severe acute encephalopathy, magnesium sulfate treatment was associated with a 36% reduction in 28-day mortality and a 43% reduction in in-hospital mortality [21]. Despite its clinical benefits, the molecular mechanisms underlying Mg-mediated neuroprotection, particularly during early brain development, remain incompletely understood.

To investigate the effects of Mg on the nervous system, researchers have employed animal models, which have provided insights into the mechanisms underlying neurological and behavioral disorders in Mg-deficient conditions [22,23]. Moreover, in animal models of Alzheimer’s disease, Mg supplementation has been shown to be synaptoprotective [5]. In rats exposed to traumatic brain injury, Mg therapy has shown a dose-dependent effect in improving cognitive performance, particularly in working memory [24]. Mg administration has also shown potential in reducing the severity of cerebral edema in animal models after stroke [25].

However, the applicability of these findings to humans is often limited due to significant differences between animal and human brains [26]. Three-dimensional brain organoids, derived from human cells, offer a more relevant platform for studying human diseases and testing potential therapies and could revolutionize drug discovery and treatment strategies. They are increasingly used as potential alternatives to animal experimentation in neuroscience research and seem to bridge the gap between patient studies and animal models [27]. In particular, incorporating BBB-like components into organoid systems enhances their ability to recapitulate neurodevelopmental processes and responses to external compounds [28]. Indeed, the endothelial component of the BBB releases significant amounts of BDNF, with obvious implications for neurodevelopmental and neurodegenerative research. Accordingly, BBB-integrated human brain organoids exhibited improved structural organization, suggesting enhanced neuronal differentiation [28]. In this context, differences in Mg bioavailability between inorganic salts such as MgSO_4_ and organic forms such as magnesium pidolate (MgPid) may be functionally relevant. We investigated the effects of MgSO_4_ and MgPid at two concentrations (1 mM, which is physiological, and 5 mM) on cortical organization and neurotransmitter systems. Through integrated morphological, ultrastructural, and molecular analyses, we show that elevated Mg levels enhance cortical layering and neuronal organization while modulating NMDA and GABA receptor expression. These findings provide new mechanistic insight into Mg-dependent regulation of cortical development and support its potential relevance for neurodevelopmental disorders and therapeutic strategies.

## 2. Materials and Methods

### 2.1. Development of Human Cerebral Organoids in the Presence of an In Vitro Model of the Blood–Brain Barrier (BBB)

Human brain organoids were generated from healthy human induced pluripotent stem cells (iPSCs, Thermo Fisher Scientific, Waltham, MA, USA) as described [29]. The iPSCs were purchased (Gibco, Thermo Fisher Scientific, Waltham, MA, USA) and cultured with mTeSR medium (Stem Cell Technologies, Vancouver, BC, Canada) in 6-well plates pre-coated with Matrigel^®^ hESC-Qualified Matrix (Corning, NY, USA). For embryoid body (EB) formation, 9000 cells/well were seeded into ultra-low-attachment 96-well plates (Corning) in 150 µL of EB medium supplemented with 4 ng/mL basic fibroblast growth factor (bFGF, Thermo Fisher Scientific) and 10 µM ROCK inhibitor Y-27632 (Sigma-Aldrich, St. Louis, MO, USA). After 7 days, EBs were transferred to a low-attachment 24-well plate (Corning) into a neural induction medium for 4 days to induce neuroectoderm formation. Immature organoids were then embedded in 15 µL droplets of Matrigel, transferred to a 6-well plate in differentiation medium, and placed on an orbital shaker to enhance nutrient and oxygen exchange, promote uniform exposure of the organoids to culture medium components, and support their growth and maturation. After 4–5 days, the culture was switched to a differentiation medium supplemented with vitamin A for 21 days. Cerebral organoids obtained after 35 days of differentiation were subsequently cultured for a further 4 days in the presence of an in vitro BBB model generated using a Transwell system with 0.4 µm inserts, as described in [13]. Briefly, the underside of each insert was coated with poly-L-lysine and seeded with human astrocytes, while the upper surface was coated with fibronectin and later seeded with human brain microvascular endothelial cells (HBMECs). After allowing astrocytes to adhere, inserts were transferred to culture plates and maintained for 72 h before endothelial cell seeding. The co-culture was then maintained for an additional 3 days, at which point the BBB reached maximal resistance and was ready for organoid co-culture. Following the establishment of the BBB, mature cerebral organoids were introduced into the lower Transwell chamber and co-cultured for 4 days.

### 2.2. Treatment with Mg Salts

Two magnesium salts were selected for treating BBB organoids (BBB-ORG): MgSO_4_, a widely used inorganic salt, and Mg pidolate (MgPid), an organic salt with higher bioavailability. Both were applied at 1 mM (physiological) and 5 mM (a relatively high concentration widely used in experimental studies to produce measurable biological effects) for 4 days. Mg-free MEM was supplemented with each salt to achieve the desired concentrations and further enriched with the appropriate growth factors for either HBMECs or mature organoids. In the Transwell system, the upper chamber contained medium with 1 or 5 mM Mg, while the lower chamber was maintained at physiological Mg levels.

### 2.3. Light and Transmission Electron Microscopy (TEM) on Resin-Embedded Organoids

BBB-ORGs were fixed in 2.5% buffered glutaraldehyde, post-fixed in 1% osmium tetroxide buffered solution, and embedded in epoxy resin (Embed 812 kit for electron microscopy, EMS, Hatfield, Pennsylvania, USA). Semithin resin sections were stained with toluidine blue and examined under a light microscope. Thin sections from selected regions were further stained with uranyl acetate/lead citrate before being analyzed using an EM-109 ZEISS transmission electron microscope (ZEISS, Oberkochen, Germany) equipped with a CCD-Megaview G2 camera (Olympus, Amburg, Germany) with ITEM imaging platform software (version ITEM-E-23082007).

### 2.4. Confocal Microscopy

At the end of each experiment, 3D organoids were immediately embedded in optimal cutting temperature (OCT) compound and cryosectioned at 10 μm using a Leica cryostat (CM 1900 Leica, Leica Biosystems, Wetzlar, Germany). The resulting sections were mounted on SuperFrost Plus adhesion slides (Epredia, Milan, Italy) and incubated overnight with primary antibodies. To evaluate the maturation stage of the human brain organoids, the sections were incubated with SRY-box transcription factor (SOX)2 (1:200) to identify neural progenitors, T-box brain (TBR)2 (10 µg/mL) to mark intermediate progenitors and subcortical neurons, and COUP-TF-interacting protein (CTIP)2 (1:500) to visualize the cortical layer (Thermo Fisher Scientific, Abcam, Cambridge, UK). To analyze dopamine receptors, antibodies against DRD1 (1:1000) and DRD2 (1:500) (Thermo Fisher Scientific) were applied. To further analyze brain receptors, NMDA-R (1:100), GABA_A_-R (1:100) (Thermo Fisher Scientific), and GABA_B_-R (10 ug/mL) (Abnova, Taipei, Taiwan) antibodies were used. Alexa Fluor 488 (green, 1 μg/mL) and 546 (red, 4 μg/mL) (Thermo Fisher Scientific) were used as secondary antibodies. Nuclei were visualized with DAPI. Images were acquired with a 40× objective in oil using an SP8 confocal microscope (Leica Microsystems, Buffalo Grove, IL, USA). Fluorescence analysis was performed with ImageJ software, version 1.54f. Data are presented as the mean of three independent experiments.

### 2.5. Western Blot

For protein extraction, pools of three organoids were lysed in buffer containing 50 mM Tris–HCl (pH 7.4), 150 mM NaCl, 1% NP-40, 0.25% sodium deoxycholate, and protease inhibitors (10 µg/mL leupeptin, 10 µg/mL aprotinin, 1 mM PMSF). Then, 50 µg per lane was resolved by SDS–PAGE and transferred to nitrocellulose membranes [29]. A Western blot was performed with primary antibodies against NMDA-R (5 μg/mL), GABA_A_-R (1:1000), dopamine receptors D1 (DRD1, 1:1000) and D2 (DRD2, 1:1000), cyclin and CBS domain divalent metal cation transport mediator 2 (CNNM2, 1:1000), transient receptor potential melastatin-subfamily member 7 (TRPM7, 1:1000) (Invitrogen, Thermo Fisher Scientific), GABA_B_-R (10 ug/mL, Abnova, Taipei, Taiwan), and TRPM6 (1:200, Santa Cruz Biotechnology, Dallas, TX, USA). β-actin (1:200, Santa Cruz Biotechnology) served as a loading control. After washing, membranes were incubated with HRP-conjugated secondary antibodies (GE Healthcare, Chicago, IL, USA), and immunoreactive bands were detected using Clarity™ ECL substrate (Bio-Rad, Hercules, CA, USA). Images were acquired on a ChemiDoc MP system (Bio-Rad), and densitometric quantification was performed using ImageJ software on at least three independent experiments. Representative blots are shown.

### 2.6. Intraorganoid Mg^2+^ Quantification

The QuantiChrom Magnesium Assay Kit (BioAssay Systems, Hayward, CA, USA) was used to measure intracellular ionized Mg (Mg^2+^) [30]. Pools of 3 organoids were lysed in PBS through repeated freezing–thawing cycles. Then, 30 µg of each sample was used to determine Mg^2+^ concentration following the manufacturer’s instructions (Supplementary Materials). Three independent experiments were performed in triplicate.

### 2.7. Measurement of GABA, Glutamate and Dopamine Levels

Intraorganoid levels of GABA and dopamine were quantified using human ELISA kits (GABA: Aviva Systems Biology, San Diego, CA, USA; dopamine: Cusabio, Houston, TX, USA), following the manufacturers’ protocols (Supplementary Materials). The assays were conducted on protein extracts derived from three pooled BBB-ORGs. The resulting colorimetric signals were measured using the Varioskan LUX Multimode Microplate Reader (Thermo Fisher Scientific).

The Glutamate Assay Kit (Cell Biolabs, San Diego, CA, USA) was employed to measure intraorganoid glutamate concentrations, following the manufacturer’s protocol (Supplementary Materials). Briefly, a pool of three organoids was lysed in PBS and freeze–thawed. Then, 20 µg/well was combined in a 1:1 ratio with the reaction mix in a 96-well black plate (Costar, Sigma-Aldrich) and incubated for 45 min at room temperature in the dark. Fluorescence intensity was measured using a microplate reader (Varioskan Lux, Thermo Fisher Scientific, Waltham, MA, USA) with an excitation wavelength of 530 nm and an emission wavelength of 600 nm. Three independent experiments were performed in triplicate.

### 2.8. Statistical Analysis

Data are presented as mean ± standard deviation (SD). Experiments were conducted at least three times, each in triplicate. Normality was assessed using the Shapiro–Wilk test, which indicated that the data were normally distributed. Statistical analysis was performed using one-way ANOVA for single-factor comparisons or two-way ANOVA for multifactorial comparisons. *p*-values from multiple comparisons were corrected using the Tukey method. Statistical significance was considered at *p*-value ≤ 0.05 (* *p* ≤ 0.05; ** *p* ≤ 0.01; *** *p* ≤ 0.001).

## 3. Results

### 3.1. Cortical Layering in BBB-ORGs Exposed to Physiological and High Mg: Morphological and Immunofluorescence Analyses

**Figure 1:**
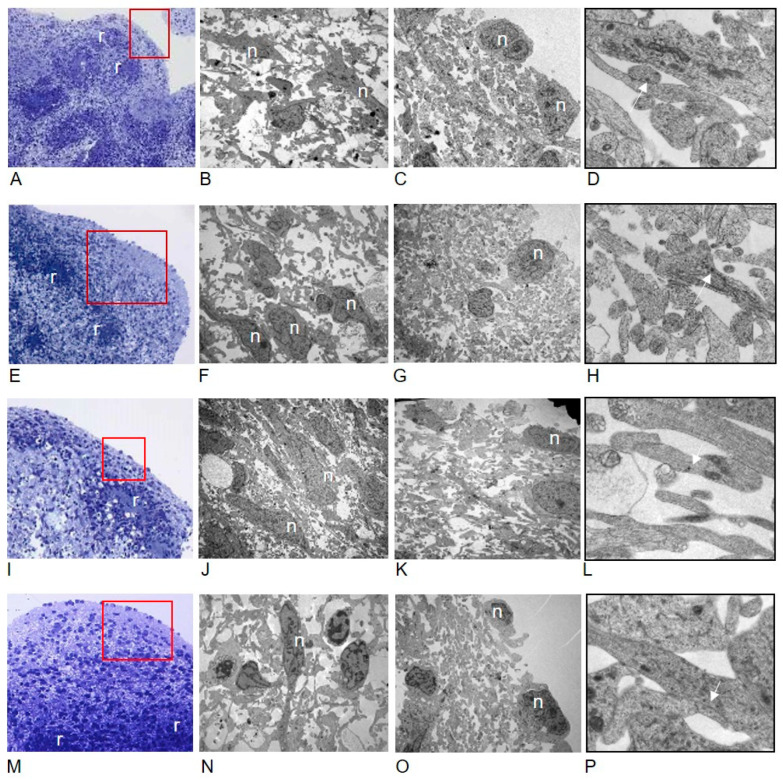
Structural and ultrastructural features of BBB-ORGs treated with MgSO_4_ or MgPid. Brain organoids cultured in the presence of a blood–brain barrier, treated with MgSO_4_ 1 mM (**A**–**D**) and 5 mM (**E**–**H**) or MgPid 1 mM (**I**–**L)** and 5 mM (**M**–**P**). Semithin sections (**A**,**E**,**I**,**M**, red boxes) highlight cortical areas overlying neuro-epithelial rosettes (r) composed of layers rich in cell bodies and an outer layer with few cells; the latter appears more prominent at higher Mg concentrations (**E**,**M**). Ultrastructural analysis of the cortical areas shows that neural cells (n) are more numerous in the deeper cortex (**B**,**F**,**J**,**N**) and are characterized by voluminous nuclei and neurites. The outermost layers are composed mainly of neurites delimited by the neural cells lining the organoid (**C**,**G**,**K**,**O**). Neurites are best recognized at higher magnification by their microtubule bundles and scattered electron-dense core vesicles (arrows) (**D**,**H**,**L**,**P**).

Light microscopy (LM) on blue-stained semithin sections (Figure 1A,E,I,M) showed organized cortical layers (Figure 1, red boxed areas) overlying neuroepithelial rosettes (Figure 1 -r-), a radial arrangement of elongated neural stem cells. Rosettes are thought to recapitulate the early stages of neural development in vivo and in brain organoids [31,32]. The above cortical layers include cell-rich layers and layers with few cells, with the latter mainly located in the outermost regions. The outer layer is poor in cell bodies and appears more prominent in organoids treated with 5 mM Mg salts compared to those exposed to 1 mM. Ultrastructural analyses showed numerous neural cells (n) (Figure 1B,F,J,N), with voluminous nuclei and well-developed neurites, mainly in the deeper cortex, and demonstrated that the outermost layers are composed of abundant neural processes (Figure 1C,D,G,H,K,L,O,P), corresponding to active neuritogenesis. Overall, what we observed suggests that the increase in thickness of the outer layer in organoids exposed to higher Mg concentrations, highlighted by LM on semithin sections, corresponds to increased neurite outgrowth, a prerequisite for neural network development in the brain organoids.

**Figure 2:**
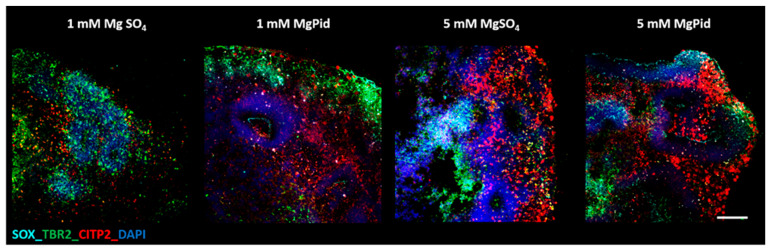
Cortical organization of BBB-ORGs treated with MgSO_4_ or MgPid. Brain organoids were cultured with 1 mM or 5 mM of MgSO_4_ or MgPid with the BBB. Immunofluorescence staining was conducted to evaluate cortical organization using antibodies against SOX2 (light blue) to identify neural progenitors, TBR2 (green) to label subcortical neurons, and CTIP2 (red) to detect the cortical neurons. DAPI (blue) was used to label nuclei. Representative images were acquired with a 20× objective. Scale bar: 100 µm.

Immunofluorescence analysis was then performed on BBB-ORG to assess the maturation stage of human brain organoids (Figure 2). SOX2 is used to identify neural progenitors [33], TBR2 to label intermediate progenitors and subcortical neurons [34], and CTIP2 to visualize the cortical layer [34]. Immunofluorescence analysis corroborates the findings obtained through electron microscopy, demonstrating distinct cortical organization in BBB-ORGs treated with physiological Mg concentrations (1 mM Mg salts, control) compared to those treated with higher concentrations. Specifically, BBB-ORGs treated with 1 mM Mg salts show an absence of proper cortical stratification, with TBR2 localized more externally and CTIP2 positioned internally. In contrast, organoids exposed to 5 mM Mg under the BBB display improved cortical organization, with CTIP2 localized in the outermost layer and TBR2 in the inner layer. This spatial arrangement suggests enhanced cortical development and maturation in organoids treated with higher Mg concentrations, indicative of a more advanced differentiation stage.

### 3.2. Mg^2+^ Levels in BBB-ORGs Exposed to Physiological and High Mg

**Figure 3:**
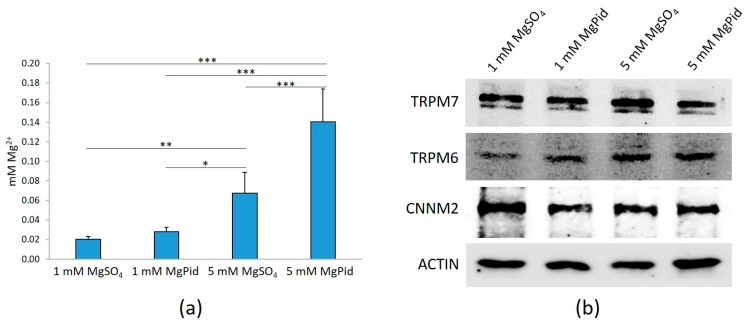
Analysis of intraorganoid Mg^2+^ and Mg transporters in BBB-ORGs treated with MgSO_4_ or MgPid. (**a**) Intraorganoid Mg^2+^ was analyzed using the QuantiChrom probe. (**b**) The levels of Mg channels/transporters TRPM7, TRPM6 and CNNM2 were analyzed by Western blot. A representative blot is shown. * *p* ≤ 0.05; ** *p* ≤ 0.01; *** *p* ≤ 0.001.

We then measured Mg^2+^ in the organoids and found it increased after incubation with 5 mM Mg salts (Figure 3a). Specifically, 5 mM MgPid was more efficient than 5 mM MgSO_4_ in increasing intraorganoid Mg^2+^ (Figure 3a). We then evaluated the total amounts of some Mg channels/transporters, i.e., TRPM6, TRPM7 and CNNM2. Western blot revealed no significant differences in protein levels in BBB-ORGs exposed to 1 or 5 mM MgSO_4_ or MgPid (Figure 3b).

### 3.3. Glutamate and N-Methyl-D-Aspartate Receptor in BBB-ORGs Exposed to Physiological and High Mg

**Figure 4:**
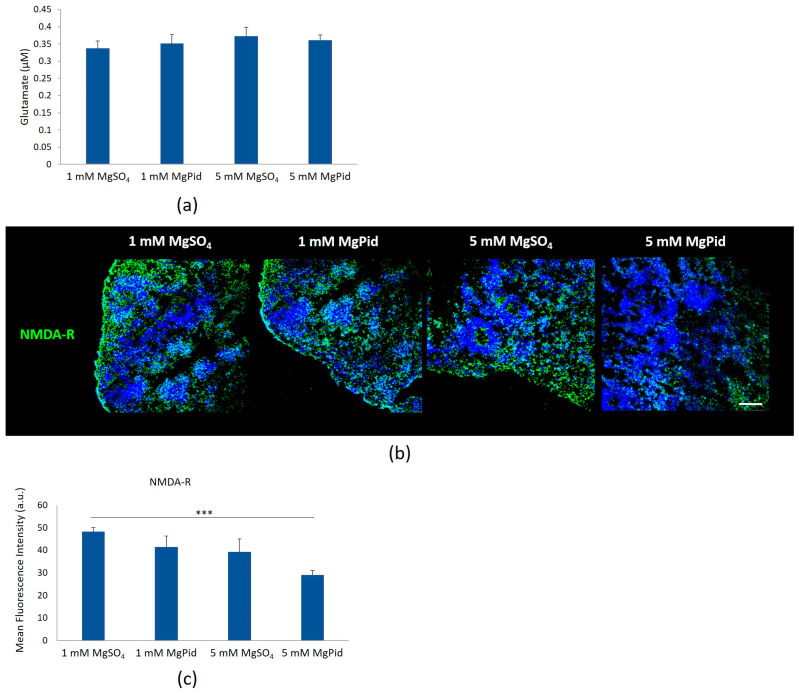
Analysis of NMDA-R and glutamate modulation in BBB-ORGs treated with MgSO_4_ or MgPid. (**a**) Brain organoids treated with 1 mM or 5 mM MgSO_4_ or MgPid under the BBB were analyzed for glutamate levels. The results are expressed as concentration (µM) ± SD. (**b**) Immunofluorescence staining was performed using antibodies against NMDA-R (green) to assess receptor distribution and DAPI (blue) to visualize nuclei. Representative images were acquired with a 20× objective. Scale bar: 100 µm. (**c**) Analysis of NMDA-R fluorescence from confocal microscopy images was performed using ImageJ. Data are presented as the mean of three independent experiments. Statistical analysis was performed using one-way ANOVA. *** *p* ≤ 0.001.

To investigate the mechanisms underlying the enhanced cortical development and maturation observed in BBB-ORGs treated with high Mg concentrations, we focused on key receptors and their corresponding neurotransmitters involved in neurodevelopment, neuroplasticity, and neuronal survival. Among these, the N-methyl-D-aspartate receptor (NMDA-R), one of the main excitatory receptors in the CNS, plays a pivotal role in synaptic plasticity and neural circuit formation [35]. Since glutamate is the primary neurotransmitter acting on NMDA-R, we quantified glutamate levels in BBB organoids treated with 1 and 5 mM MgSO_4_ or MgPid and found no significant differences (Figure 4a).

We then performed immunofluorescence staining for NMDA-R. The receptor appeared reduced in organoids treated with 5 mM MgPid (Figure 4b,c), consistent with the results obtained by Western blot analysis (Figure S1).

### 3.4. GABA and Its Receptors in BBB-ORGs Exposed to Physiological and High Mg

**Figure 5:**
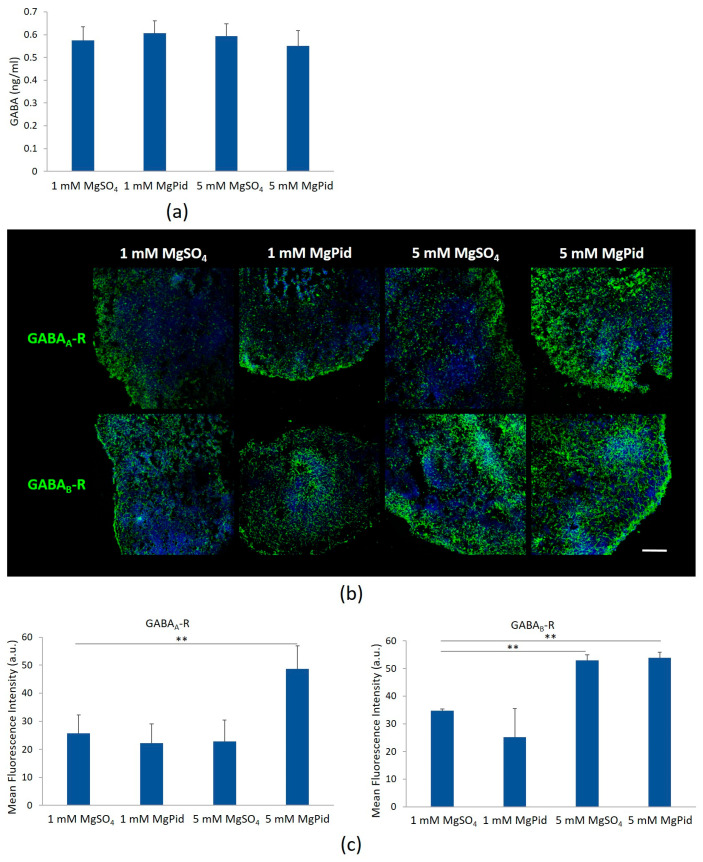
Analysis of GABA_A_-R, GABA_B_-R and GABA modulation in BBB-ORG treated with MgSO_4_ or MgPid. (**a**) Brain organoids treated with 1 mM or 5 mM MgSO_4_ or MgPid under the BBB were analyzed for GABA levels by ELISA assay. (**b**) Immunofluorescence staining was performed using antibodies against GABA-Rs (green) to assess receptor distribution and DAPI to visualize nuclei (blue). Representative images are acquired with a 20× objective. Scale bar: 100 µm. (**c**) Analysis of GABA_A_-R and GABA_B_-R fluorescence from confocal microscopy images was performed using ImageJ. Data are presented as the mean of three independent experiments. Statistical analysis was performed using one-way ANOVA. ** *p* ≤ 0.01.

GABA is an inhibitory neurotransmitter involved in neurodevelopment [36]. In BBB-ORGs maintained in 1 or 5 mM MgSO_4_ or MgPid, we did not detect any difference in the total amounts of GABA by ELISA (Figure 5a). Immunofluorescence staining was performed for GABA_A_-R and GABA_B_-R. Higher levels of both receptors were observed in organoids treated with high Mg concentrations (Figure 5b,c) compared to those treated with physiological Mg. Specifically, GABA_A_-R is significantly upregulated in BBB organoids treated with 5 mM MgPid, while GABA_B_-R is enhanced in organoids treated with both 5 mM MgPid and 5 mM MgSO_4_, consistent with the results obtained from Western blot analysis (Figure S2).

### 3.5. Dopamine and Its Receptors in BBB-ORGs Exposed to Physiological and High Mg

**Figure 6:**
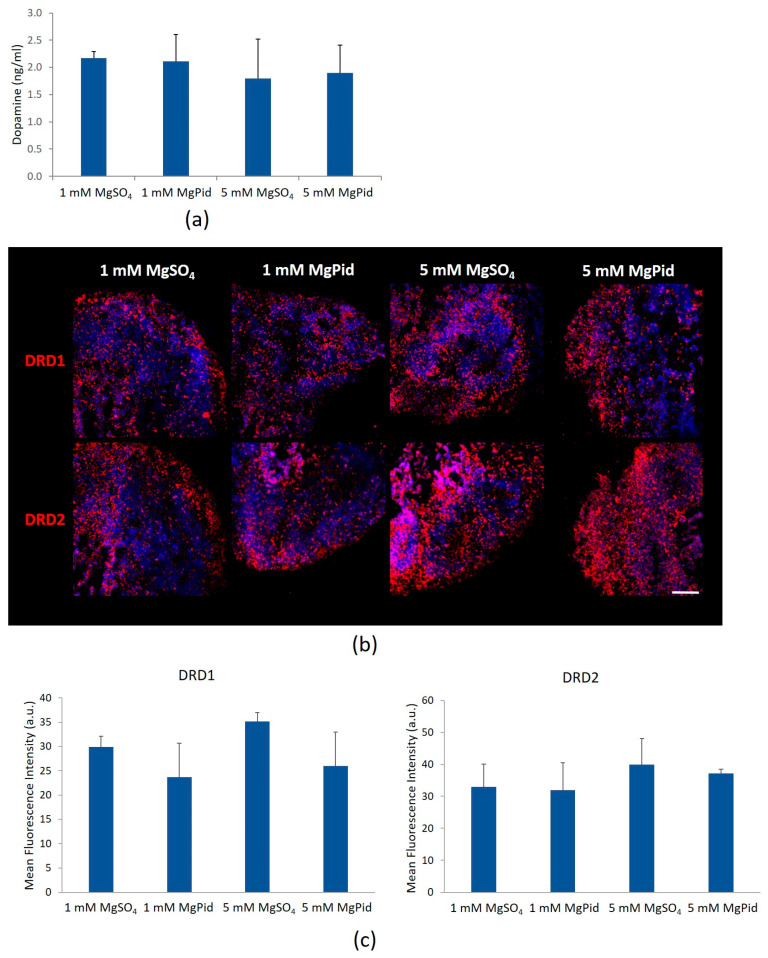
Analysis of DRD1, DRD2 and dopamine modulation in BBB-ORG treated with MgSO_4_ or MgPid. (**a**) Brain organoids treated with 1 mM or 5 mM MgSO_4_ or MgPid under the BBB were analyzed for dopamine levels by ELISA assay. (**b**) Immunofluorescence staining was performed using antibodies against DRDs (red) to detect the receptors and DAPI to visualize nuclei (blue). Representative images are acquired with a 20× objective. Scale bar: 100 µm. (**c**) Analysis of DRD fluorescence from confocal microscopy images was performed using ImageJ. Data are presented as the mean of three independent experiments. Statistical analysis was performed using one-way ANOVA.

Dopamine and its receptors, DRD1 and DRD2, play essential roles in neural development, synaptic plasticity, and cortical maturation [37]. To assess whether Mg supplementation affects the dopaminergic system in BBB-ORGs, we analyzed dopamine concentrations as well as the levels and localization of DRD1 and DRD2 in BBB organoids exposed to 1 or 5 mM MgSO_4_ or MgPid (Figure 6). ELISA analysis revealed no significant differences in dopamine levels across all samples (Figure 6a). Immunofluorescence staining showed no notable changes in the localization of DRD1 and DRD2 between treatments with 1 or 5 mM Mg salts (Figure 6b,c). Similarly, Western blot analysis indicated that the total levels of DRD1 and DRD2 remained unchanged (Figure S3).

## 4. Discussion

Our results demonstrate that Mg supplementation enhances cortical organization and neuronal differentiation in BBB-ORGs. Morphological and ultrastructural analyses revealed a pronounced thickening of cortical layers, together with more elaborate neurite-like structures in organoids exposed to 5 mM compared to 1 mM Mg salts. These results align with previous studies highlighting Mg’s essential role in neurogenesis, synaptic plasticity, and neuronal maturation [10,38]. Immunofluorescence analyses further support these observations, showing that higher Mg concentrations promote more clearly defined cortical layers with appropriate localization of key neuronal markers. Specifically, TBR2 is a transcription factor that serves as a key marker for identifying and studying intermediate progenitor cells, which are vital for the generation of glutamatergic neurons in key brain regions like the cerebral cortex and hippocampus [39,40]. CTIP2 is another transcription factor that plays a role in orchestrating the development of cortical layers and the establishment of functional neural circuits [41,42]. Taken together, the enhanced cortical stratification observed in Mg-supplemented BBB-ORGs suggests that Mg may facilitate neuronal differentiation and migration, potentially through its modulation of intracellular signaling pathways important in cortical development [43], independent of the specific Mg salt employed.

Our data show that high Mg significantly modulates neurotransmitter receptors, particularly NMDA receptors—a subtype of glutamate receptors involved in synaptic transmission, learning, and memory—as well as GABA receptors, which mediate inhibitory signaling essential for neuronal circuit formation. The balance between excitatory glutamatergic signaling via NMDA-R and inhibitory GABAergic signaling is fundamental to synaptic plasticity and brain development [44], and disruptions in this equilibrium are implicated in various neurological disorders [45]. In our model, glutamate levels remained stable across treatments, yet NMDA-R expression was significantly downregulated in organoids exposed to 5 mM MgPid. Conversely, both ionotropic GABA_A_ and metabotropic GABA_B_ receptors were significantly upregulated with 5 mM Mg levels, particularly with MgPid, while GABA levels themselves were unchanged. These results suggest that Mg enhances inhibitory tone in developing neuronal networks [46], potentially contributing to the structural improvements observed in cortical layering. The differential effects between MgPid and MgSO_4_ imply that Mg salts vary in their cellular uptake or downstream signaling effects, warranting further study.

Although dopamine and its receptors DRD1 and DRD2 contribute to neurogenesis, synaptic plasticity, and cortical development [47], Mg supplementation did not significantly alter dopamine levels or receptor expression in our organoids. This indicates that the primary effects of 5 mM Mg on BBB-ORGs are mediated via glutamatergic and GABAergic pathways rather than dopaminergic signaling. While Mg-related modulation of dopamine has been reported in fetal rodent models [48], our results suggest that such effects may be less prominent in human-derived organoids.

The markedly increased intraorganoid Mg^2+^ levels following 5 mM MgPid treatment likely contribute to the more pronounced molecular effects observed under this condition. Although both MgSO_4_ and MgPid improved cortical organization, the downregulation of NMDA-R and the significant upregulation of GABA_A_-R were specific to high MgPid exposure, suggesting a unique capacity of this organic Mg salt to modulate neurotransmitter receptor homeostasis.

Multiple families of Mg transporters, with several members expressed in the brain, likely play specific roles in maintaining optimal Mg levels across different cell types and compartments. The highly homologous proteins TRPM6 and TRPM7, possessing both ion channel and kinase domains, are important in Mg homeostasis and contribute to brain function and development [49]. CNNM2 is ubiquitously expressed in mammalian tissues, with particularly high levels in the kidney, brain, and lung [50]. Notably, mutations in the CNNM2 gene have been implicated in the etiology of certain forms of intellectual disability and seizures [51,52], and genome-wide association studies have identified CNNM2 as a robust risk gene for schizophrenia [53]. TRPM6, TRPM7, and CNNM2 expression remained unchanged across conditions in BBB-ORGs. Intracellular Mg can increase without changes in CNNM, TRPM6, or TRPM7 expression because Mg homeostasis is regulated not only at the transcriptional level but also through channel activity, post-translational regulation, intracellular buffering, and Mg efflux. TRPM7 activity, for example, is tightly regulated by intracellular Mg^2+^ and Mg-ATP levels, suggesting that altered transporter function rather than expression may underlie increased intracellular Mg [54]. More studies are needed to clarify this mechanism.

Our results underscore the translational potential of targeting brain Mg homeostasis as a multimodal strategy to combat the pathological progression of neurodegenerative diseases. Elevated central Mg concentrations exert robust neuroprotective effects primarily by modulating the N-methyl-D-aspartate (NMDA) receptor complex; here, Mg serves as a voltage-dependent antagonist that prevents glutamate-induced excitotoxicity and subsequent neuronal apoptosis [1]. Beyond receptor blockade, optimized Mg levels enhance synaptic plasticity and density, reinforcing the structural foundations of cognitive resilience. Centrally, magnesium mitigates chronic neuroinflammation by suppressing microglial activation and the subsequent release of pro-inflammatory cytokines. This is complemented by its role in preserving the integrity of the blood–brain barrier (BBB), which prevents the infiltration of systemic neurotoxic substances into the parenchyma [1,55]. These multi-targeted mechanisms, including the optimization of mitochondrial efficiency to reduce oxidative stress, collectively contribute to the preservation of cognitive function and the attenuation of pathology in neurodegenerative disorders such as Alzheimer’s and Parkinson’s diseases. The clinical validation of these mechanisms is supported by recent evidence; a 2026 randomized controlled trial demonstrated that supplementation with magnesium L-threonate significantly improved overall cognitive performance and reduced “estimated brain cognitive age” by 7.5 years in adults [56]. Consequently, maintaining brain Mg homeostasis represents a critical therapeutic target for ameliorating age-related cognitive decline and neuroinflammatory progression.

Future research should prioritize elucidating the precise mechanisms through which Mg influences neurotransmitter receptor expression and neural differentiation, as well as investigating its potential long-term effects on synaptic function and neural network stability. Moreover, to apply our findings for the therapeutic management of neurological disorders using Mg, it will be important to evaluate the effects of Mg deficiency in organoids prior to treatment.

This study has several limitations that warrant consideration. First, all experiments were conducted on brain organoids at a single time point (35 days of culture). Consequently, these data provide a snapshot of a specific stage rather than a longitudinal view; analyzing organoids across multiple developmental stages could offer deeper insights into the temporal dynamics of protein expression. In addition, although human brain organoids represent a valuable and physiologically relevant in vitro model, they do not fully recapitulate the architectural or functional complexity of the developing human brain in vivo. While the integration of an in vitro BBB model significantly enhances physiological relevance, it remains a reductive system that cannot entirely mimic the dynamic interactions occurring in vivo.

## 5. Conclusions

Our findings in brain organoids carry implications for understanding the neurodevelopmental roles of Mg. They underscore the potential of Mg supplementation as a modulator of neural development and highlight its relevance for therapeutic strategies targeting neurodevelopmental disorders characterized by disruptions in cortical organization or excitatory–inhibitory balance. Future research should prioritize elucidating the precise mechanisms through which Mg influences neurotransmitter receptor expression and neural differentiation, as well as investigating its potential long-term effects on synaptic function and neural network stability.

## Data Availability

The data presented in this study are openly available in Dataverse through the following link: https://dataverse.unimi.it/dataverse/MagnesiumSupplementation (accessed on 24 May 2026).

## Supplementary Materials

The following supporting information can be downloaded at https://www.mdpi.com/article/10.3390/biomedicines14061242/s1, Materials and Methods: 2.6. Intraorganoid Mg^2+^ Quantification; 2.7. Measurement of GABA, glutamate and dopamine levels. Figure S1. Analysis of NMDA-R in BBB-ORGs treated with MgSO_4_ or MgPid. NMDA-R expression was analysed by Western blot. A representative blot (left panel) and densitometry (right panel) are shown, with actin used as a loading control to ensure equal protein loading across samples. Statistical analysis was performed using one-way ANOVA. ** *p* ≤ 0.01. Figure S2. Analysis of GABAA-R and GABAB-R modulation in BBB-ORG treated with MgSO_4_ or MgPid. GABA_A_-R, GABA_B_-R expression was analysed by Western blot. A representative blot (left panel) and densitometry (right panel) are shown, with actin used as a loading control to ensure equal protein loading across samples. Statistical analysis was performed using one-way ANOVA. ** *p* ≤ 0.01; *** *p* ≤ 0.001. Figure S3. Analysis of DRD1 and DRD2 modulation in BBB-ORG treated with MgSO_4_ or MgPid. DRD1 and DRD2 expression was analysed by Western blot. A representative blot (left panel) and densitometry (right panel) are shown, with actin used as a loading control to ensure equal protein loading across samples. Statistical analysis was performed using one-way ANOVA.

## Abbreviations

The following abbreviations are used in this manuscript:

Mg	magnesium
BBB	blood–brain barrier
BBB-ORG	BBB organoids
CNS	central nervous system
NMDA	N-methyl-D-aspartate
GABA_A_-R	gamma-aminobutyric acid type A receptor
GABA_B_-R	gamma-aminobutyric acid type B receptor
BDNF	brain-derived neurotrophic factor (BDNF)
MgPid	magnesium pidolate
DRD1	dopamine receptor D1
DRD2	dopamine receptor D2
CNNM2	cyclin and CBS domain divalent metal cation transport mediator 2
TRPM7	transient receptor potential melastatin-subfamily member 7
TRPM6	transient receptor potential melastatin-subfamily member 6
HBMECs	human brain microvascular endothelial cells
TBR2	T-box brain 2
CTIP2	COUP-TF-interacting protein 2
