# Supplementary material for: Magnesium Supplementation Improves Cortical Stratification and Neuronal Differentiation in Blood–Brain Barrier-Integrated Human Brain Organoids
Source: Biomedicines. 2026 May 29;14(6):1242. doi: 10.3390/biomedicines14061242 (PMC13297601; doi:10.3390/biomedicines14061242)
Supplement: Supplementary file 1 [file biomedicines-14-01242-s001.zip › biomedicines-4316565-supplementary.pdf]

# Magnesium Supplementation Improves Cortical Stratification and Neuronal Differentiation in Blood-Brain Barrier-integrated Human Brain Organoids

**Sara Castiglioni, Antonella Tosoni, Manuela Nebuloni, Jeanette A. Maier**

## Materials and Methods

*2.6. Intraorganoid Mg2+ Quantification*

QuantiChrom Magnesium Assay

Samples and standard (2 mg/dL) were incubated with the working reagent for 2 min, and the optical density was read at 500 nm (OD for samples and standard). After the addition of 10 µL of EDTA solution, the optical density was measured again at 500 nm (OD for blanks). The magnesium concentration in the sample was calculated as follows:

((ODsample – ODblank)/ ODmg – OdmgBlank) X 2 (mg/dL)

*2.7. Measurement of GABA, glutamate and dopamine levels*

GABA ELISA

The Aviva Systems Biology GABA ELISA Kit is based on a competitive enzyme immunoassay. Microplate wells are pre-coated with an anti-GABA antibody, and samples or standards are added together with a fixed amount of biotinylated GABA. The endogenous GABA competes with the labeled GABA for binding sites on the antibody. After washing away unbound material, avidin–HRP is added, followed by TMB substrate to produce a colorimetric reaction. The reaction is stopped with an acidic solution, and absorbance is measured at 450 nm. The signal is inversely proportional to the GABA concentration in the sample.

Dopamine ELISA

Standards and samples were added to the wells (in duplicate for standards), followed by the addition of HRP-conjugate and antibody, while a blank well was included without reagents. After incubation at 37°C for 1 h, the plate was washed thoroughly three times to remove unbound components. Subsequently, substrate solutions A and B were added and the plate was incubated for 15 min at 37°C in the dark. The reaction was then stopped with stop solution, and absorbance was measured at 450 nm.

Glutamate Assay

Fifty microliters of each standard or sample were dispensed into a black fluorescence microtiter plate, followed by the addition of 50 µL of reaction mix. After thorough mixing, the plate was incubated for 30–45 min at 37°C, protected from light. Since the assay is continuous, fluorescence can be measured at multiple time points. The plate was then read using a fluorescence microplate reader (excitation 530–570 nm, emission 590–600 nm), and glutamate concentrations were determined by comparison with a standard curve.

# Figures

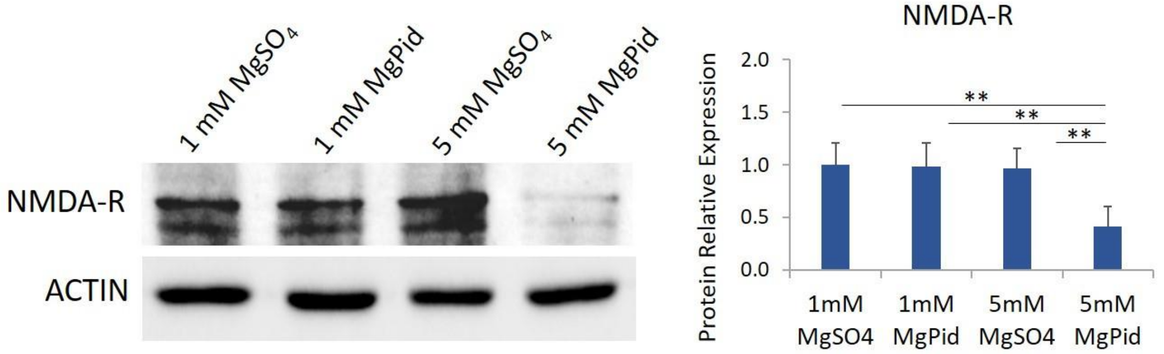


**Figure S1.** Analysis of NMDA-R in BBB-ORGs treated with $MgSO_4$ or MgPid. NMDA-R expression was analysed by Western blot. A representative blot (left panel) and densitometry (right panel) are shown, with actin used as a loading control to ensure equal protein loading across samples. Statistical analysis was performed using one-way ANOVA. ** $p \leq 0.01$.

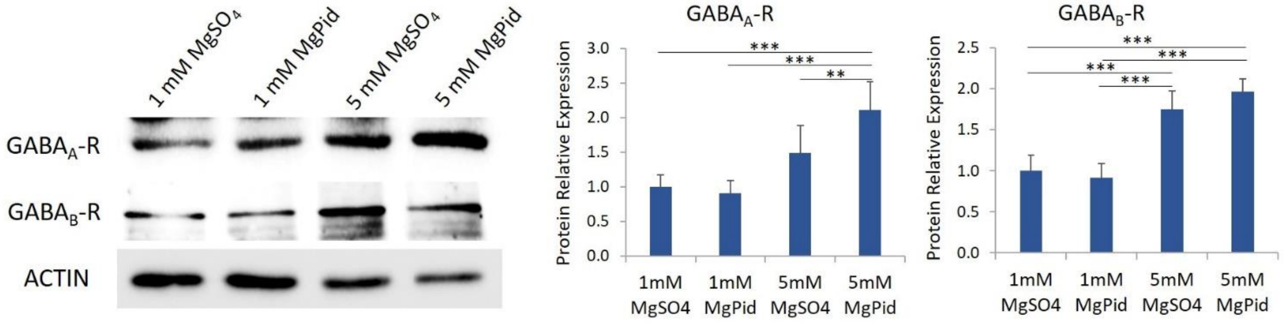


**Figure S2.** Analysis of $GABA_A$-R and $GABA_B$-R modulation in BBB-ORG treated with $MgSO_4$ or MgPid. $GABA_A$-R, $GABA_B$-R expression was analysed by Western blot. A representative blot (left panel) and densitometry (right panel) are shown, with actin used as a loading control to ensure equal protein loading across samples. Statistical analysis was performed using one-way ANOVA. ** $p \leq 0.01$; *** $p \leq 0.001$.

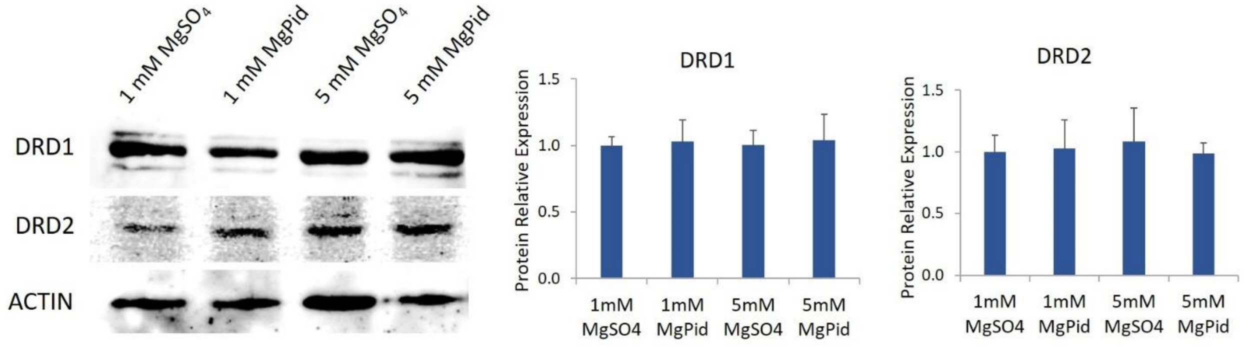


**Figure S3.** Analysis of DRD1 and DRD2 modulation in BBB-ORG treated with $MgSO_4$ or MgPid. DRD1 and DRD2 expression was analysed by Western blot. A representative blot (left panel) and densitometry (right panel) are shown, with actin used as a loading control to ensure equal protein loading across samples. Statistical analysis was performed using one-way ANOVA.
